# 3-year clinical outcomes of *A S*ingapore *V*enaSeal™ real world post-market evaluation *S*tudy (*ASVS*) for varicose vein ablation

**DOI:** 10.1186/s42155-024-00452-8

**Published:** 2024-04-27

**Authors:** Tjun Yip Tang, Charyl Jia Qi Yap, Sze Ling Chan, Shereen Xue Yun Soon, Vanessa Bao Xian Khoo, Edward Choke, Tze Tec Chong

**Affiliations:** 1 The Vascular & Endovascular Clinic, Gleneagles Medical Centre, 6 Napier Road #08-08, Sinagpore, 258499 Singapore; 2 Department of Vascular Surgery, Singapore General Hospital, Singapore, Singapore; 3 Health Services Research Center, SingHealth, Singapore, Singapore; 4 Department of General Surgery, Sengkang General Hospital, Singapore, Singapore

**Keywords:** VenaSeal™ closure system, Endovenous, Cyanoacrylate closure, Varicose vein, Chronic venous insufficiency

## Abstract

**Introduction:**

Medium-term clinical outcome data are lacking for cyanoacrylate glue (CAG) ablation for symptomatic varicose veins, especially from the Asian population.

**Objectives:**

Aim was to determine the 3-year symptomatic relief gained from using the VenaSeal™ device to close refluxing truncal veins from the Singaporean ASVS prospective registry.

**Methods:**

The revised Venous Clinical Severity Score (rVCSS) and three quality of life (QoL) questionnaires were completed to assess clinical improvement in venous disease symptoms along with a dedicated patient satisfaction survey. 70 patients (107 limbs; 40 females; mean age of 60.9 ± 13.6 years) were included at 3 years.

**Results:**

At 3 years, rVCSS showed sustained improvement from baseline (5.00 to 0.00; *p* < 0.001) and 51/70 (72.9%) had improvement by at least 2 or more CEAP categories.

Freedom from reintervention was 90% and 85.7% patients were extremely satisfied with the treatment outcome. No further reports of further hypersensitivity reactions after one year.

**Conclusion:**

The 3-year follow-up results of the ASVS registry demonstrated continued and sustained clinical efficacy with few reinterventions following CAG embolization in Asian patients with chronic venous insufficiency.

**Trial registration:**

ClinicalTrials.gov Registration: NCT03893201.

## Introduction

*A Singapore VenaSeal™ real world post-market evaluation Study (ASVS)* evaluated the clinical efficacy of the VenaSeal™ Closure System (VSCS) (Medtronic, Dublin, Ireland) to ablate symptomatic refluxing truncal veins. ASVS showed that the technology was safe in 100 Asian patients and was associated with high efficacy in terms of truncal closure, technical success and patient satisfaction at the 2 week, 3- and 12- month intervals [1, 2]. However, the UK *National Institute of Health and Clinical Excellence* (NICE) currently still recommend thermal ablation as first-line endovenous treatment modalities for symptomatic superficial truncal saphenous vein incompetence [3] despite the fact that there is now level 1 evidence from the US VeClose RCT showing non-inferiority of the VSCS compared to radiofrequency ablation, in terms of successful incompetent great saphenous vein (GSV) occlusion and symptom improvement was sustained to 5 years [4]. The aim was to report 3 year clinical outcomes from the ASVS registry.

## Methods

*ASVS* was a real-world, prospective, single arm, multi-centre, multi-investigator trial investigating the use of VSCS in a cohort of multi-ethnic Asian patients with symptomatic chronic venous insufficiency and varicose veins from Singapore. Study design, inclusion, exclusion criteria, procedural and peri-operative care protocols and outcomes through 12 months have been previously described [1]. Patients of Clinical, Etiology, Anatomy and pathophysiology (CEAP) classification 2–5 were included. Ethical approval was gained from the Institution Review Board of both centres and informed consent was gained from all participants.

Outcome of interest was clinical improvement at 3 years, assessed on the basis of change in revised Venous Clinical Severity Score (rVCSS) and CEAP. In addition, patients completed 3 quality of life surveys—EuroQol-5 Dimension questionnaire (EQ-5D), Aberdeen Varicose Vein Questionnaire (AVVQ) and Chronic Venous Insufficiency quality life Questionnaire-14 (CIVIQ-14) [5]. Occurrence, severity of any further adverse events and reinterventions were documented. Patients completed a brief questionnaire about treatment satisfaction and whether they would have the operation again if required to, which has been previously described in detail [1]. Unfortunately, due to the COVID-19 pandemic, we were unable to perform Duplex ultrasound confirmation of anatomical closure, unless there was a clinical need for potential reintervention.

## Results

Seventy out of the original one hundred patients were included (40 (57.1%) females, 107 limbs, 68 (63.6%) bilateral GSV ablation) with a mean age of 66.4 ± 11.9 years. Majority were Chinese (51/70; 72.9%). Patient demographics/ clinical variables are summarized in Table 1. As previously reported, there was 100% technical success rate and no device-related complications during truncal vein embolization.

**Table 1:** ASVS baseline characteristics

**Characteristic**	**Number of subjects at Baseline** (*n* = *100*)	**Number of subjects at 12 m** (*n* = *90)*	**Number of subjects at 36 m** (*n* = *70)*
**Gender**			
Male	41	37 (41.1)	30 (42.9)
Female	59	53 (58.9)	40 (57.1)
**Mean age**, years (SD)	60.1 ± 12.8	60.7 ± 12.8	60.9 ± 13.6
**Mean BMI**, kg/m^2^ (SD)	26.7 ± 4.58	26.6 ± 4.65	26.2 ± 4.32
**Ethnic Group**			
Chinese	71	64 (71.1)	51 (72.9)
Malay	11	9 (10.0)	8 (11.4)
Indian	16	16 (17.8)	10 (14.3)
Others	2	1 (1.1)	1 (1.43)
**Smoking**			
Smoker	9	6 (6.7)	4 (5.71)
Non-Smoker	84	78 (86.7)	63 (90.0)
Former Smoker	7	6 (6.7)	3 (4.29)
**Primary Symptoms**			
Pain	37	32 (35.6)	24 (34.3)
Aching	43	33 (36.7)	25 (35.7)
Swelling	57	52 (57.8)	39 (55.7)
Heaviness	46	45 (50.0)	29 (41.4)
Burning	2	1 (1.1)	0 (0.00)
Itch	26	24 (26.7)	14 (20.0)
Others	30	4 (4.4)	22 (31.4)
**Comorbidities**			
Hypertension	45	40 (44.4)	31 (44.3)
Hyperlipidemia	37	33 (36.7)	24 (34.3)
Diabetes Mellitus	16	14 (15.6)	11 (15.7)
Ischemic Heart Disease	6	5 (5.6)	4 (5.71)
**CEAP category**			
C2 (varicose veins)	24	20 (22.2)	15 (21.4)
C3 (edema)	33	30 (33.3)	25 (35.7)
C4a (pigmentation/eczema)	32	28 (31.1)	21 (30.0)
C4b (lipodermatosclerosis)	4	4 (4.4)	3 (4.29)
C5 (healed venous ulcer)	7	7 (7.8)	3 (4.29)
**Duration of Varicose Veins** (months), median (IQR)	24.00 (7.75–60.0)	24.00 (7.00–60.0)	24.0 (6.00 – 60.0)
**Distribution of Truncal Endovenous Ablation**	(*n* = 151 legs)	(*n* = 138 legs)	(*n* = 107 legs)
GSV	49 (32.5%)	41 (29.7%)	35 (32.7)
Bilateral GSV	96 (63.6%)	92 (66.7%)	68 (63.6)
SSV	1 (0.7%)	1 (0.7%)	1 (0.93)
Combined unilateral GSV and SSV or ATV	5 (3.3%)	4 (2.9%)	3 (2.80)
**Total number of truncal veins treated**	(*n* = 156 veins)	(*n* = 142 veins)	(*n* = 110veins)
GSV	150 (96.2)	137 (96.5)	106 (96.4)
SSV	5 (3.2)	4 (2.82)	4 (3.64)
ATV	1 (0.6)	1 (0.7)	0 (0.0)

**Fig. 1:**
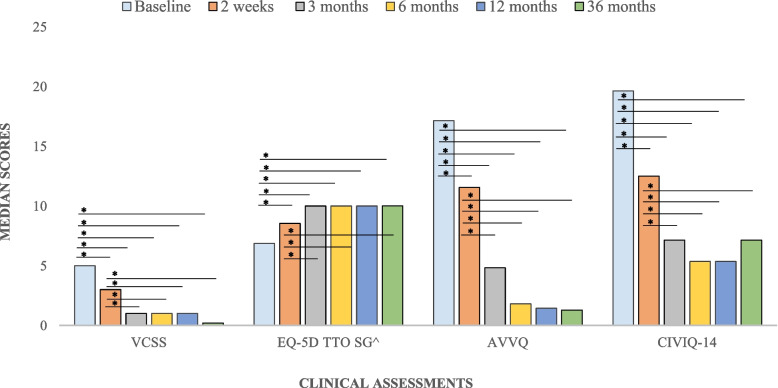
^ scaled up by 10 for presentation purposes. *p < 0.05

The median follow-up period was 41.5 (IQR 38.8 – 43.9) months. Median rVCSS showed sustained improvement from baseline through to 36 months (5.00 to 0.00; *p* < 0.001) (Table 2). Figure 1 and Table 2 summarize rVCSS, AVVQ, CIVIC-14 and EQ-5D scores at baseline, 2 weeks, 3, 6,12 and 36 months visits. Improvement in all four measures was statistically significant and sustained between baseline and at all timepoints (*p* < 0.001).

**Table 2:** Follow-up clinical assessments

Assessments	Median (IQR)	*P* value (baseline –2 weeks/3/6/12/36 months)	*P* value (2 weeks – 3/6/12/36 months)	*P* value (3 – 6/12 /36 months)	*P* value (6 – 12/36 months)	*P* value (12–36 months)
**VCSS**						
**Baseline**	5.00 (4.00–7.00)	-	-	-	-	-
**2 weeks**	3.00 (2.00–5.00)	< 0.001^*^	-	-	-	-
**3 months**	1.00 (0.00–3.00)	< 0.001^*^	< 0.001^*^	-	-	-
**6 months**	1.00 (0.00 – 2.00)	< 0.001^*^	< 0.001^*^	0.910	-	-
**12 months**	1.00 (0.00 – 3.00)	< 0.001^*^	< 0.001^*^	0.369	0.938	-
**36 months**	0.00 (0.00 – 1.00)	< 0.001^*^	< 0.001^*^	0.999	0.782	0.256
**EQ-5D TTO SG**						
**Baseline**	0.686 (0.430 – 0.890)	-	-	-	-	-
**2 weeks**	0.854 (0.700 – 1.00)	< 0.001^*^	**-**	**-**	**-**	**-**
**3 months**	1.00 (0.854 – 1.00)	< 0.001^*^	0.0015^*^	**-**	**-**	**-**
**6 months**	1.00 (0.854 – 1.00)	< 0.001^*^	0.018^*^	**0.985**	**-**	**-**
**12 months**	1.00 (0.890 – 1.00)	< 0.001^*^	0.006^*^	**0.999**	**0.945**	**-**
**36 months**	1.00 (0.838 – 1.00)	< 0.001^*^	**0.404**	**0.553**	**0.902**	**0.413**
**AVVQ**						
**Baseline**	17.1 (11.1–25.4)	-	-	-	-	-
**2 weeks**	11.6 (5.90–19.3)	0.0004^*^	**-**	**-**	**-**	**-**
**3 months**	4.83 (0.00–9.78)	< 0.001^*^	< 0.001^*^	**-**	**-**	**-**
**6 months**	1.81 (0.00–6.68)	< 0.001^*^	< 0.001^*^	**0.651**	**-**	**-**
**12 months**	1.45 (0.00–10.7)	< 0.001^*^	< 0.001^*^	**0.653**	**1.00**	**-**
**36 months**	1.29 (0.00 – 5.39)	< 0.001^*^	< 0.001^*^	**0.579**	**0.999**	**0.999**
**CIVIQ-14**						
**Baseline**	19.64 (12.05–28.57)	-	-	-	-	-
**2 weeks**	12.50 (7.14–16.96)	< 0.001^*^	**-**	**-**	**-**	**-**
**3 months**	7.14 (0.00–13.39)	< 0.001^*^	< 0.001^*^	**-**	**-**	**-**
**6 months**	5.357 (0.00–12.05)	< 0.001^*^	< 0.001^*^	**0.910**	**-**	**-**
**12 months**	5.357 (0.00–10.71)	< 0.001^*^	< 0.001^*^	**0.369**	**0.939**	**-**
**36 months**	7.14 (0.00–10.71)	< 0.001^*^	0.0002^*^	**0.999**	**0.782**	**0.255**

*VCSS* Venous Clinical Severity Score, *EQ-5D* EuroQol-5 Dimension, *TTO* time trade-off, *AVVQ* Aberdeen Varicose Vein Questionnaire, *CIVIQ-14* Chronic Venous Insufficiency quality life Questionnaire-14

^*^Significant at *p* < *0.05* when compared to baseline values

There was a significant improvement of CEAP score at 3 years compared to baseline (median 1 (IQR 0–2) from 3 (IQR 3–4); *p* < 0.05) and 51/70 (72.9%) had improvement by at least 2 or more CEAP categories (Table 3).

**Table 3:** Changes in CEAP classification

	**Baseline (*n* = 100)**	**3 Years (*n* = 70)**
**CEAP Classification**		
C0 (Asymptomatic)	0	33 (47.1)
C1 (Reticular Veins)	0	12 (17.1)
C2 (Varicose Veins)	24	8 (11.4)
C3 (Edema)	33	5 (7.1)
C4 (Skin Changes)	36	11 (15.7)
C5 (Healed venous ulcer)	7	1 (1.4)
**Median CEAP (IQR)**	3 (3 – 4)	1 (0 – 2)
**Improvement in CEAP**		
Worsened by ≥ 1 category	-	4 (5.7)
Unchanged	-	10 (14.3)
Improved by ≥ 2 category	-	51 (72.9)

There were no further hypersensivity or phlebitic episodes reported after one year (Table 4). 4/70 (5.7%) and 13/70 (18.6%) developed new and recurrent symptoms respectively. However, only 3 (4.2%) patients required reintervention between the 1–3 year timepoints (one deep vein interrogation and iliac vein stenting for non thrombotic iliac vein compression syndrome and 2 patients for progression of below the knee great saphenous vein (GSV) reflux). Overall freedom from reintervention at three – years was 63/70 (90.0%). We had previously reported 8/90 (8.9%) patients who complained of a pulling sensation during walking or exercise between the 3 and 12 months follow-up visits because of the fibrosed GSV cord located just below the skin in predominantly thin females, following CAG embolization. By 3 years, only 2/70 (2.9%) were still reporting this symptom. At 3 years, 60/70 (85.7%) were very or extremely satisfied with their treatment outcome and 62/70 (88.6%) would probably or definitely recommend this type of treatment to their next of kin or friend if required (Table 5).

**Table 4:** Adverse events & reinterventions

Adverse events	12 months (*n* = 90)	36 months (*n* = 70)
**Allergic Skin Reaction (Redness/itch)**	0 (0.0)	0 (0.0)
**Pulling Sensation**	8 (8.9)	2 (2.9)
**Newly Developed**		
Heaviness	3 (3.3)	2 (2.9)
Swelling	1 (1.1)	1 (1.4)
Hyperpigmentation	1 (1.1)	1 (1.4)
**Recurrence of symptoms**		
Varicosities	0 (0.0)	3 (4.3)
Heaviness	0 (0.0)	3 (4.3)
Swelling	3 (3.3)	7 (10.0)
Ulcers	0 (0.0)	0 (0.0)
**Further Interventions for CVI**	5 (5.6)	7 (10.0)
Deep Vein Interrogation	4 (80.0)	4 (57.1)^a^
Mean time to Deep Vein Interrogation procedure, months (± SD)	7.6 ± 3.2	11.4 ± 7.6
Superficial Vein Ablation	1 (20.0)	3 (42.9)^b^
Mean Time to SVA procedure, months (± SD)	7.1 (NA)	17.6 ± 1.0

^a^One new IVUS/stenting procedure between 1–3 years timepoints

^b^Two new below the knee GSV ablation for new symptomatic reflux

**Table 5:** Patient satisfaction

Satisfaction criteria	Percentage of subjects n(%)
**Extremely/Very Satisfied**	
**Baseline**	-
**2 weeks**	66/100 (66.0)
**3 months**	72/91 (79.0)
**6 months**	74/90 (82.2)
**12 months**	78/90 (86.7)
**36 months**	60/70 (85.7)
**Definitely/Probably recommend**	
**Baseline**	-
**2 weeks**	76/100 (76.0)
**3 months**	79/91 (87.0)
**6 months**	79/90 (87.8)
**12 months**	81/90 (90.0)
**36 months**	62/70 (88.6)
**Appearance much/somewhat improved**	
**Baseline**	-
**2 weeks**	69/100 (69.0)
**3 months**	60/91 (66.0)
**6 months**	59/90 (65.6)
**12 months**	70/90 (77.8)
**36 months**	58/70 (82.9)
**Symptoms much/somewhat improved**	
**Baseline**	-
**2 weeks**	73/100 (73.0)
**3 months**	83/91 (91.0)
**6 months**	86/90 (95.5)
**12 months**	85/90 (94.4)
**36 months**	64/70 (91.4)

## Discussion

The ASVS registry showed a sustained clinical efficacy and patient satisfaction after VenaSeal™ ablation through 3 years. There were few reinterventions since one year and the worrying pulling phenomenon prevalence we had reported previously had reduced. These data are in keeping with the 3-year efficacy and safety results from the *first-in-human* use of cyanoacrylate glue for GSV incompetence [6], the European multicentre eScope registry [7] and the 5-year US VeClose RCT [4], which all assessed the utility of the VSCS for varicose veins and the only studies to date with medium term results published. Our study has the advantage of recruiting a purely Asian cohort albeit with no Duplex-defined anatomical closure confirmation of the axial vein at the three year time-point because of the COVID-19 crisis, which led to many patients being unable to come back for their dedicated follow-up. However, it does reinforce the sustained clinical effect and improvement of QoL of CAG closure at the medium term in Asian CVI patients. The reintervention rate was low (< 5%) and the majority were for new below the knee GSV reflux, which had not been present at the initial Duplex scan when the patients had first enrolled into the study. It was also encouraging that there were no reinterventions for recanalizations. The persistent clinical benefit was further indicated by continued patient satisfaction scores (> 85%). Those who were not extremely satisfied or would not happily recommend the procedure did not complain of any adverse events related to the procedure such as a hypersensitivity reaction or thrombophlebitis but were more neutral and not on the extreme opposite end because of new or recurring leg symptoms requiring further imaging or investigations. The pulling sensation rate of the fibrotic cord created after CAG embolization of the more superficial GSV in thin women seen in 8.9% patients at the one year follow-up had reduced at 3 years (2.9%). It is thought that CAG does not produce significant thrombosis because the vein walls are immediately coapted to the medical adhesive by the application of external compression resulting in an inflammatory and eventual fibrotic reaction rather than a thrombotic one [8]. This may well delay the fibrotic reaction leading to the patient still feeling the “inflamed” GSV longer than if the truncal vein were blocked using a thermal ablation technique.

Limitations of the study include 30% of patients were unable to be contacted during this time and lack of truncal occlusion data by Duplex ultrasound because of the COVID-19 crisis making it difficult for research patients to come to the hospital for imaging. We plan to address this when we perform 5-year ASVS data analysis.

## Conclusion

The 3-year follow-up results of the ASVS registry demonstrated continued and sustained clinical efficacy with few reinterventions following CAG embolization in Asian patients with chronic venous insufficiency.

## Data Availability

The datasets generated during the current study are not publicly available due to Personal Data Protection Act (PDPA).

## Abbreviations

AVVQ: Aberdeen Varicose Vein Questionnaire
CAG: Cyanoacrylate Glue
CEAP: Clinical-Etiology-Anatomy-Pathophysiology Classification
CIVIQ: Chronic Venous Insufficiency Quality of Life Questionaire
CVI: Chronic Venous Insufficiency
EQ5D: EuroQol-5 Dimension questionnaire
GSV: Great Saphenous Vein
QoL: Quality of Life
rVCSS: Revised Venous Clinical Severity Score
